# Association of differential miRNA expression with hepatic vs. peritoneal metastatic spread in colorectal cancer

**DOI:** 10.1186/s12885-018-4043-0

**Published:** 2018-02-20

**Authors:** Sabine Heublein, Markus Albertsmeier, David Pfeifer, Lisa Loehrs, Alexandr V. Bazhin, Thomas Kirchner, Jens Werner, Jens Neumann, Martin Kurt Angele

**Affiliations:** 1 Department of General, Visceral, Transplantation and Vascular Surgery, University Hospital LMU Munich, Marachioninistrasse 15, 81377 Munich, Germany; 2 Department of Obstetrics and Gynaecology, Heidelberg University Hospital, Heidelberg, Germany; 3 Institute of Pathology, Ludwig-Maximilians-Universität München, Munich, Germany; 4 German Cancer Consortium (DKTK), Heidelberg, Germany; 5 German Cancer Research Center (DKFZ), Heidelberg, Germany

**Keywords:** Colorectal cancer, miRNA, Metastasis

## Abstract

**Background:**

Though peritoneal carcinomatosis reflects a late stage of colorectal cancer (CRC), only few patients present with synchronous or metachronous liver metastases alongside their peritoneal carcinomatosis. It is hypothesized that this phenomenon may be causally linked to molecular characteristics of the primary CRC. This study used miRNA profiling of primary CRC tissue either metastasized to the liver, to the peritoneum or not metastasized at all thus to identify miRNAs potentially associated with defining the site of metastatic spread in CRC.

**Methods:**

Tissue of the primary tumor stemming from CRC patients diagnosed for either liver metastasis (LM; *n* = 10) or peritoneal carcinomatosis (PER; n = 10) was analyzed in this study. Advanced CRC cases without metastasis (M0; *n* = 3) were also included thus to select on those miRNAs most potentially associated with determining metastatic spread in general. miRNA profiling of 754 different miRNAs was performed in each group. MiRNAs being either differentially expressed comparing PER and LM or even triple differentially expressed (PER vs. LM vs. M0) were identified. Differentially expressed miRNAs were further validated by in silico and functional analysis.

**Results:**

Comparative analysis identified 41 miRNAs to be differentially expressed comparing primary tumors metastasized to the liver as opposed to those spread to the peritoneum. A set of 31 miRNAs was significantly induced in primary tumors that spread to the peritoneum (PER), while the remaining 10 miRNAs were found to be repressed. Out of these 41 miRNAs a number of 25 miRNAs was triple-differentially expressed (i.e. differentially expressed comparing LM vs. PER vs. M0). The latter underwent in silico analysis. Finally, we demonstrated that miR-31 down-regulated c-MET in DLD-1 colon cancer cells.

**Conclusions:**

This study demonstrates that CRC primary tumors spread to the peritoneum vs. metastasized to the liver display significantly different miRNA profiles. Larger patient cohorts will be needed to validate whether determination of e.g. miR-31 may aid to predict the course of disease and whether this may help to create individualized follow up or treatment protocols. To determine whether certain miRNAs may be involved in regulating the metastatic potential of CRC, functional studies will be essential.

## Background

Metastatic spread is regarded a common feature of colorectal cancer (CRC). About 20% of CRC cases present with distant metastasis even at the time of primary diagnosis [1]. Approximately 60% of patients diagnosed for advanced staged CRC is estimated to develop distant metastasis within 5 years [2]. Liver metastases account for about one third of all distant metastasis locations in CRC [3]. Another common site of distant spread is the peritoneum and about one out of four patients develops peritoneal carcinomatosis during the course of disease [4]. Importantly, although peritoneal carcinomatosis mirrors a late stage of disease, only few patients present with synchronous or metachronous hematogenous i.e. liver metastases [5, 6]. Our recent work highlighted that stem cell features of the primary tumor may direct the way of metastasis [5, 7]. Those CRCs that will spread to the liver display a prominent cancer stem cell-like immuno-phenotype while those which will metastasize to the peritoneum do not. We hypothesized that that primary CRC cases developing solely PC but no hematogenous metastases lack the stem cell features needed for dissemination [5].

Factors related to this phenomenon by regulating mRNA stability have not been identified yet. Though miRNAs are considered to regulate metastatic potential of various cancer cells, it remains unexplored whether miRNA are involved in directing the metastatic route in CRC. In general, miRNAs represent small RNAs that majorly contribute to post-translational gene regulation. Since miRNAs may be determined by a fully automated, high throughput procedure they may become attractive for routine diagnostic in the future. Further, since miRNAs may be counteracted by sequence specific antisense oligo-nucleotides, they may evolve to be druggable targets. We used an expression platform covering a set of highly characterized miRNAs as well as several of the more recently discovered miRNAs along with the star miR* sequences. miRNA profiling of primary tumor tissue was employed to identify those miRNAs potentially being associated with determining the location of synchronic or metachronic metastatic spread in CRC.

## Methods

### Patients

Patients diagnosed for CRC (*n* = 23) between 1988 and 2012 were included in the study. Patients were selected from a study panel published earlier [5] and were further characterized within the current analysis. All patients underwent surgical resection of their CRC at the Department of General, Visceral, Transplantation and Vascular Surgery Surgery, University Hospital Munich (Munich, Germany). Formalin-fixed-paraffin-embedded tissue of the primary tumor was used for all the analysis described. CRC tissue underwent routine histopathological processing and examination. FFPE sample were stored under standardized conditions.

Patients were classified as either pT3 (15/23) or pT4 (8/23). Most patients were graded as high (14/23) at initial diagnosis. About half of all cases were male (12/23) and were diagnosed CRC that had already spread to abdominal lymph nodes (18/23). Mean age of the cohort was 65.8 ± 12.5 years.

Patients were retrospectively grouped into three groups according to metastasis location: group M0: CRC without metastasis formation during follow up period (*n* = 3), group LM: CRC metastasized to the liver either at initial diagnosis or during follow up without peritoneal carcinomatosis (*n* = 10), group PER: CRC spread to the peritoneum either at initial diagnosis or during follow up without liver metastasis (n = 10).

### Study design

Patient data and tumor samples were retrieved from a patient panel that was collected prospectively and that has been published earlier by our group [5]. Samples were further characterized in the current analysis. The outcome assessed was metastasis formation after a mean follow up of 9.6 ± 2.2 years. During follow up 14 deaths were observed and mean overall survival was 8.4 ± 2.1 years.

### Assay methods

#### RNA isolation, processing and multiplex qRT-PCR

RNA isolation was performed on freshly sliced FFPE tissue samples. All preparation steps were performed under sterile, RNAse and DNAse free conditions. First, samples were dewaxed by xylene and washed in absolute ethanol. Representative tumor areas were extracted by microscope assisted microdissection. Dissection was supervised by an experienced senior pathologist (JN). Tumor stroma or other connective tissue was strictly excluded during sample preparation. A serial slide of each sample was H&E stained thus to ensure that areas of necrotic tumor, lymphocyte rich regions or areas directly adjacent to the invasive front do not get included. RNA was transferred into 1.5 ml tubes and further processed by applying the RecoverAll™ Total Nucleic Acid Isolation Kit for FFPE (Applied Biosystems, Carlsbad, CA) as per manufacturer’s recommendation.

Amount and quality of total RNA was quantified by a NanoDrop (Thermo Fisher Scientific, Waltham, MA) spectrophotometer. Those samples that had passed the quality control (A260/280 > 2.0, clear single RNA peak) were processed further. RNA concentrations were adjusted and equal amounts of RNA underwent cDNA synthesis using either Panel A of B multiplex primer sets (Applied Biosystems). cDNA synthesis was performed using the TaqMan® MicroRNA Reverse Transcription Kit (Applied Biosystems) according to manufacturer’s protocol. Reverse transcription was run on a Mastercycler gradient PCR machine (Eppendorf, Hamburg, Germany) using the following PCR program: (2 min at 16 °C, 1 min at 42 °C, 1 sec  at 50 °C) × 40 cycles followed by 5 min at 85 °C and cooling down to 4 °C. cDNAs were pre-amplified using miRNA specific primers as provided with the Megaplex™ Primer Pools, Human Pools Set v3.0 (Applied Biosystems). Pre-amplification was run on a Mastercycler gradient PCR machine (Eppendorf) using the following PCR program: 10 min at 95 °C, 2 min at 55 °C, 2 min at 72 °C, 12 amplification cycles (15 s at 95 °C and 4 min at 60 °C) followed by 10 min at 99.9 °C and cooling down to 4 °C.

Pre-amplified cDNA underwent single assay PCR for RNU44 and hsa-miR185-5p to check for nucleic acid integrity. Finally, equal volumes of diluted, pre-amplified cDNA, TaqMan® Universal PCR Master Mix, No AmpErase® UNG and DEPC-water were loaded onto TaqMan® Array Human MicroRNA A + B Cards Set (Applied Biosystems). Three patient samples per group (M0, LM, PER) were pooled. qRT-PCR run was performed. MiRNAs being sufficiently expressed (i.e. cycle threshold (CT) lower than 30 – as recommended by Applied Biosystems) were selected for comparative analysis. RNU44, RNU48 and U6 snRNA were available as housekeeping genes on all TLDA cards run. Ath-miR159a served as a negative control.

The 2^-ddCT^ method was used to quantify relative miRNA expression (dCT (target sample) = CT (target gene) – CT (housekeeping gene); ddCT (target sample) = dCT (target sample) – dCT (reference sample)) [8]. A set of 25 miRNAs was found to be triple differentially expressed. Out of these 25 miRNAs three miRNAs were selected for further analysis. First, the TLDA result was validated by single PCR on three cases per group (that had been measured on the TLDA card as well) and second, expression of miR-215-5p, miR-31-5p and miR-483-5p was analyzed in the whole study sample.

#### c-MET immunohistochemistry

c-MET immunohistochemistry (IHC) was performed on LM and PER samples. The staining method employed had been extensively validated and published by our group before [9]. In brief, c-MET (monoclonal anti-rabbit IgG, clone EP1454Y, Epitomics, Burlingame, CA) was used at a dilution of 1:150 and was detected using the Vectastain Elite ABC kit (Vector, Burlingame, CA). DAB (Dako, Glostrup, Denmark) served as a chromogen. System (without primary antibody) and isotype (unspecific rabbit IgG instead of the primary antibody) controls were performed to control for unspecific staining. c-MET IHC staining was determined using the H-score [9].

#### miR31 target gene validation

A human miRNA expression vector (pEZX-MR04) encoding either hsa-miR31 or a scrambled miRNA (both from Gene Copoeia, Rockville, MD) was transfected into DLD-1 cells by using Lipofectamine 2000 as a transfection system. Transfection was performed as per manufacturer’s recommendation using a 4:1 (transfection reagent to plasmid) ratio. This ratio had been demonstrated to achieve the highest transfection efficiency (data not shown). Successful transfection of the transgene and expression of miR-31 was monitored by TaqMan qRT-PCR. RNU44 served as a house-keeping gene.

#### Cell culture

DLD-1 colon cancer cells were purchased from ATCC (LGC Standards GmbH, Wesel, Germany). Cell lines were routinely checked for mycoplasma contamination and cell line authentication and were found to be contamination free. RPMI1640 containing 10% fetal calf serum was used as a standard culture media. Neither antibiotics nor anti-mycotics were added to culture media. Cells were passaged twice a week. Only those cultures passaged at least 4 times and maximum up to 15 times were used for the experiments.

#### Western blotting

Cells were seeded at a density of 75% per well and transfection was performed 24 h after plating. Plates were incubated 3 days at 37 °C in a humidified atmosphere and were afterwards lysed in RIPA buffer containing protease and phosphatase inhibitors. Lysates were cleared by centrifugation at 15.000 rpm for 15 min at 4 °C. Total protein concentrations of the supernatants were quantified and adjusted. The Mini-Protean System (Biorad, Hercules, CA) was used for polyacrylamide gel electrophoresis and blotting. PVDF membranes were blocked in 5% milk powder in TBS-0.1%Tween20 (TBST) for 1 h at room temperature. Rabbit anti-cMet (Cell Signalling Technologies; diluted 1∶1000 in 5% milk powder-TBST) and rabbit anti-GAPDH (Cell Signalling Technologies; diluted 1∶2000 in 5% milk powder-TBST) were used to incubate membranes overnight. Membranes were processed using a goat anti-rabbit IRDye® 680RD secondary antibody (Cell Signalling Technologies) according to the manufacturer’s instructions. Each experiment was repeated three times under the same conditions achieving similar results. Blots were quantified by employing the QuantityOne analysis software (Biorad, Hercules, CA).

### Statistical analysis methods

This study has been carried out according to the REMARK (Reporting Recommendations for Tumor Marker Prognostic Studies) criteria [10].

The IBM statistics package SPSS (version 22) was used to test data for statistical significance. Fisher’s exact test and Student’s T test were used. Cell culture experiments were repeated three times achieving similar results. Replicates were performed independently and on different passages of cells. A probability value of *p* < 0.05 was deemed significant in all statistical tests.

## Results

### Study cohort

The current analysis selected 23 cases from a larger study panel previously investigated by Neumann et al. [5] for miRNA analysis. The current study sample (*n* = 23) was characterized as follows: 10 patients diagnosed for CRC metastasized to the liver either at initial diagnosis or during follow up without peritoneal carcinomatosis (termed ‘LM’), 10 patients diagnosed for CRC spread to the peritoneum either at initial diagnosis or during follow up without liver metastasis (termed ‘PER’) and finally three CRC patients without metastasis formation during follow up period (termed ‘M0’). Patient characteristics and clinico-pathological variables according to WHO 2010 and TNM 2009 are summarized in Table 1. Gender, age, grade, UICC stage and pN-Stage were not significantly different when cases with peritoneal carcinomatosis and cases diagnosed for liver metastasis were compared. Those tumors that had spread to the peritoneum were more often assigned a pT4 grade than those which presented with liver metastasis (7/10 vs. 0/10; *p* = 0.003).

**Table 1:** Patients’ characteristics

	LM	PER	M0
UICC			
II	0	0	1
III	0	3	2
IV	10	7	0
Grading			
G2	6	2	1
G3	4	8	2
pT			
pT3	10	3	2
pT4	0	7	1
pN			
pN0	3	0	2
pN +	7	10	1
sex			
female	4	4	3
male	6	6	0

**Fig. 1:**
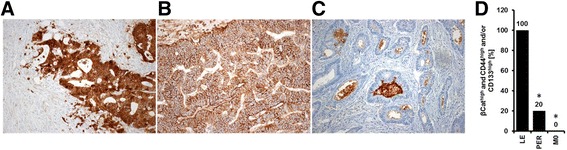
beta Catenin (**a**), CD44 (**b**) and CD133 (**c**) immunopositivity served to sub-define primary tumors that had either spread to the peritoneum or to the liver: Presence of a stem cell like phenotype as defined by beta Catenin (**a**), CD44 (**b**) and/or CD133 (**c**) high immunopositivity was found to characterize primary tumors that spread to the liver, while absence of a stem cell like immuno-phenotype was characteristic for CRC metastasized to the peritoneum [5]. The current analysis selected 23 cases (n (LM) = 10, n (PER) = 10, n (M0) = 3) from the study panel previously investigated by Neumann et al. for further characterization including miRNA analysis. Presence of a stem cell phenotype (βCat^high^ and CD44^high^ and/or CD133^high^) in LM samples and absence of this stem cell classifier in PER and M0 samples was highly reproducible in the sub-sample analyzed in the current study (**d**). The stem cell phenotype was much rarer in PER (*p* = 0.001) and M0 (*p* = 0.003) than in LM samples. Significant changes are indicated by stars (*) in (**d**)

A recent study from our group found that expression of a stem cell classifier (βCat^high^ and CD44^high^ and/or CD133^high^) in primary CRC tissue predicts metastatic spread to the liver [5], while absence of a stem cell like immuno-phenotype was characteristic for CRC metastasized to the peritoneum. Regarding the patient cohort studied in the current work, the stem cell classifier (βCat^high^ and CD44^high^ and/or CD133^high^) was detected in all those primary tumors that had set liver metastasis either at time of diagnosis or during follow up (10/10). The βCat^high^ and CD44^high^ and/or CD133^high^-phenotype was much rarer in cancers that presented alongside with peritoneal carcinomatosis (PER group, 2/10, *p* = 0.001) or had not set metastasis at all (M0 group; 0/3, *p* = 0.003) (Fig. 1).

### Differential miRNA expression is associated with metastatic spread of the primary CRC

**Fig. 2:**
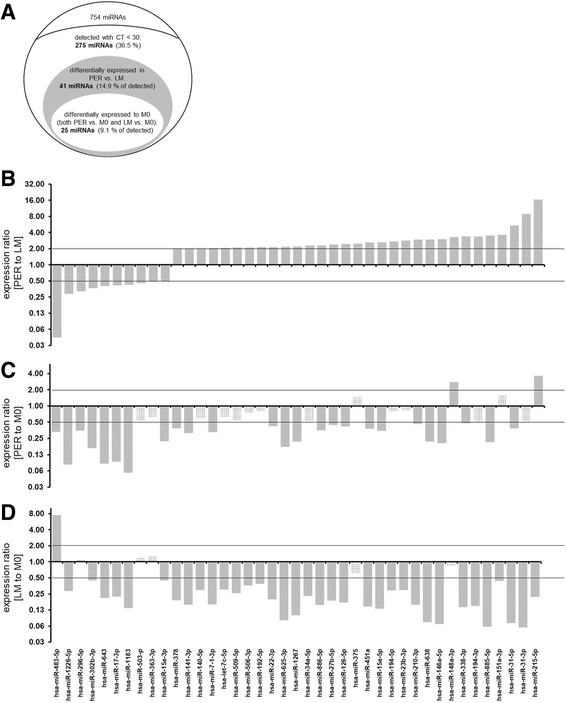
miRNAs found to be differentially expressed. miRNA analysis and selection has been summarized schematically (**a**). miRNAs found to be differentially expressed in tumors that had spread to the peritoneum as compared with those metastasized to the liver are displayed (**b**). These miRNAs differentially expressed comparing LM and PER were then normalized to expression of the respective miRNA in M0 (**c, d**). Expression levels of PER vs. M0 are shown in **c**, while expression levels of LM vs. M0 are shown in **d**. Grey charts represent significant changes whereas squared charts stand for miRNAs not differentially expressed in the respective analysis (**c**, **d**). Expression ratios (2^-ddCT^) are displayed on a log2 scale

MiRNA profiling was performed in primary colon cancer tissue of the study sample described above. Seven hundred fifty-eight miRNAs (754 target miRNAs and 4 control RNAs) were analyzed (Fig. 2a). A total number of 275 miRNAs (36.5%) was detected with a cycle threshold (CT) lower than 30 and underwent further analysis. Out of these 275 miRNAs a number of 41 miRNAs was identified to be differentially expressed (upregulated > 2.00-fold or downregulated < 0.500-fold) when cases diagnosed with liver metastasis (LM) were compared to those with peritoneal carcinomatosis (PER) (Fig. 2b). A set of 31 miRNAs was significantly up-regulated in the PER group, while 10 miRNAs were found to be repressed in PER as compared to LM. The miRNAs most significantly induced in PER were hsa-miR-215-5p (17-fold), hsa-miR-31-3p (8.9-fold) and hsa-miR-31-5p (5.4-fold). On the opposite, miR-483-5p (0.04-fold), hsa-miR-1226-5p (0.29-fold) and hsa-miR-296-5p (0.32-fold) were identified to be most obviously repressed (Fig. 2b).

As a second step, we questioned how to further select on those miRNAs potentially associated with peritoneal carcinomatosis and hence not only differentially expressed to LM but also to M0 (abbreviated as PER to M0 in Fig. 2c). A set of 27 miRNAs out of these 41 miRNAs identified previously was also found to be differentially expressed to M0 (Fig. 2c). hsa-miR-148a-3p (2.8-fold) and hsa-miR-215-5p (3.6-fold) were upregulated when normalized to M0, while the remaining 25 miRNAs were downregulated. The same analysis was repeated regarding differential expression of miRNAs in primary tumors metastasized to the liver (LM) as compared to tumors that did not metastasize at all (M0) (abbreviated as LM to M0 in Fig. 2d). A number of 36 miRNAs out of the 41 miRNAs described above (Fig. 2b) was also found to be differentially expressed regarding LM to M0 (Fig. 2d). Comparing LM to M0 only a single miRNA (hsa-miR-483-5p) was upregulated (7.5-fold).

**Fig. 3:**
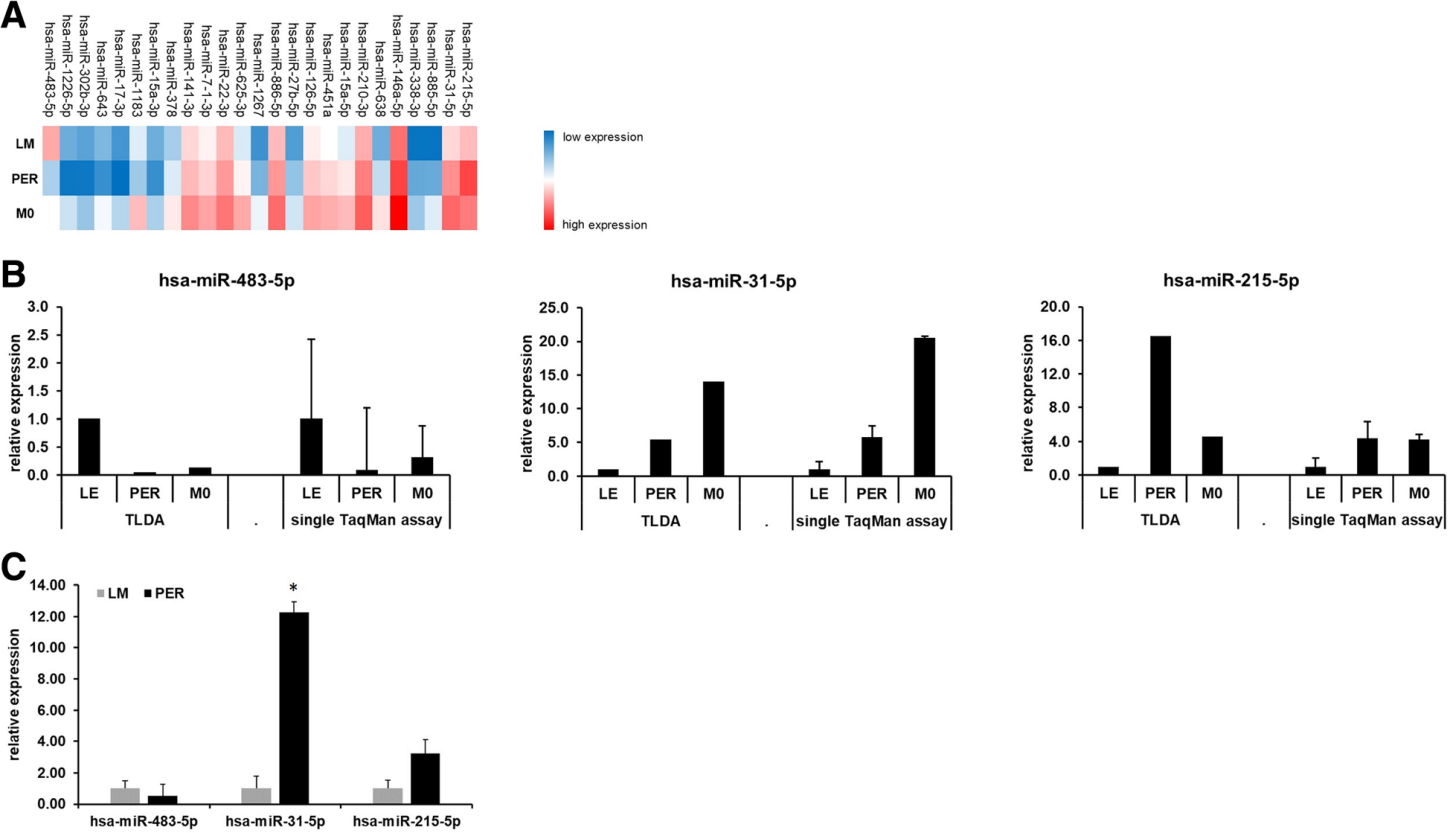
Validation of selected miRNA expression patterns. 25 miRNAs were found to be triple differentially expressed and are displayed as heat map (**a**). The heat map presents dCT data (dCT = CT (target gene) – CT (housekeeping gene)) with red indicating high (min. dCT value: - 2.4) and blue indicating low (max. dCT value: 11.1) expression (3-color scale with white representing the midpoint). Three of these genes characterized as triple differentially expressed were selected and analyzed by single TaqMan PCR assay PCR (**b**). Single PCR assays referred to in B were run on the same samples that had been analyzed on the TLDA card (n (LM) = 3, n (PER) = 3, n (M0) = 3) thus to show that the data deriving from the TLDA cards (referred to as “TLDA” in (**b**)) can be reproduced by running single assays (referred to as “single TaqMan assay” in (**b**)) on the cycler. Finally, expression of hsa-miR-483-5p, hsa-miR-31-5p and hsa-miR-215-5p was analyzed in the whole study cohort (n (LM) = 10, n (PER) = 10, **c**). Error bars represent standard error of mean. Significant changes in **c** (*p* < 0.05) are indicated with stars (*)

Taken together comparative analysis of PER vs. LM (Fig. 2b), PER vs. M0 (Fig. 2c) and LM vs. M0 (Fig. 2d) revealed a set of 25 miRNAs to be triple-differentially expressed, i.e. differentially among PER vs. LM, PER vs. M0 and LM vs. M0 at the same time (Fig. 3a). These miRNAs underwent in silico prediction analysis for their association to colorectal cancer. According to the miRCancer search algorithm [11–14] out of these 25 miRNAs being triple-differentially expressed a number of 16 miRNAs had been published to be linked to CRC in the past. Out of these hsa-miR-215-5p, hsa-miR-31-5p as well as hsa-miR-483-5p were further validated by single PCR. Results obtained from TLDA cards were found to be highly reproducible on single PCR level (Fig. 3b). hsa-miR-215-5p, hsa-miR-31-5p and hsa-miR-483-5p were then analyzed on the whole patient sample. Again, hsa-miR-483-5p was repressed (0.51-fold) and both hsa-miR-215-5p - (3.2-fold) and hsa-miR-31-5p (12-fold) were induced (Fig. 3c). Statistical analysis comparing miRNA expression on the whole sample only proved a significant difference in case of hsa-miR-31-5p (*p* = 0.002; Fig. 3c).

### miR-31 reduces c-MET expression in DLD-1 colon cancer cells

We finally questioned whether hsa-miR-31-5p may functionally interfere with metastasis formation. A literature search revealed that hsa-miR-31-5p has been reported to regulate genes involved in epithelial-to-mesenchymal transition (EMT) [15–18], which is assumed to be a major step within metastasis formation [19]. For instance, Mitamura et al. reported miR-31 to directly target c-MET – a prominent mediator of EMT - in an ovarian cancer model [15]. We thus selected c-MET to test whether miR-31 is active in colon cancer cells.

**Fig. 4:**
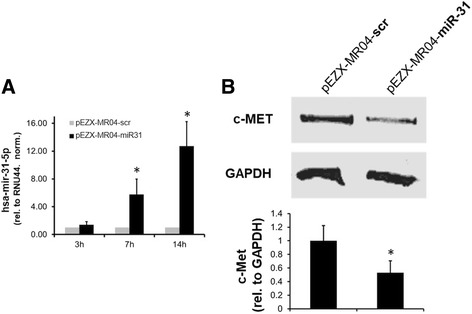
Target gene regulation by miR-31. Expression of hsa-miR-31-5p in DLD-1 cells transfected with miR-31- or scr-control plasmid was monitored 3, 7 and 14 h after transfection by TaqMan-PCR (**a**). In addition, c-Met protein was determined 14 h after transfection by western blotting (**b**). Stars (*) indicate significant changes (*p* < 0.05). Data derive from three different experiments achieving similar results

DLD-1 colon cancer cells were transfected with pEZX-MR04-miR-31 or pEZX-MR04-scr control vector. Target gene expression was monitored by qRT-PCR 3, 7 and 14 h after transfection. Although we observed a net increase in hsa-miR-31-5p expression at all the time points analysed (3 h: 1.4-fold, 7 h: 5.8-fold, 12 h: 13-fold), statistical testing proved significant induction for the 14 h (*p* = 0.013) and the 7 h incubation period (*p* = 0.046) (Fig. 4a). As a consequence, protein expression of c-MET was measured 14 h after transfection and western blotting revealed significant downregulation of c-MET (0.53-fold, *p* < 0.001; Fig. 4b).

Interestingly, regarding the patient panel expression of hsa-miR-31-5p was inversely correlated (*p* = 0.011) with presence of the stem cell classifier (βCat^high^ and CD44^high^ and/or CD133^high^). This is consistent with the observation that the stem cell classifier has been detected in all those cancers spread to the liver which at the same time are expressing hsa-miR-31-5p on a low level (Fig. 3c). On the other hand, PER cases were negative for the stem cell classifier while abundantly expressing hsa-mir-31-5p (Figs. 1d and 3c). Although c-MET was regulated by miR-31 in DLD-1 colon cancer cells, we could not identify a significant statistical association of hsa-miR-31-5p expression and c-MET immunopositivity in the patients’ tumor tissue samples.

## Discussion

### Determination of metastatic spread in colorectal cancer

Several reports hypothesize that the risk of distant metastatic spread may be predicted from the molecular profile of the primary tumor [5, 20]. As a future perspective, biomarkers identified in the primary tumor may aid to forecast the course of disease i.e. location of metastatic spread or whether metastasis will develop at all. Such attempt may one day aid the clinician to plan individualized follow up and potentially treatment protocols.

The probability of distant spread and poor outcome has been linked to a 17-gene signature identified across different types of human solid tumors [20]. Apart from predicting the probability of metastatic spread in general, molecular characteristics of the primary tumor may also forecast the site of metastasis formation [5, 21]. Neumann et al. found that this phenomenon may be related to the cancer stem cell phenotype of the primary tumor and that those primary CRCs that lack stem cell like characteristics may less often set liver metastasis during their clinical course [5]. Potentially, this may be linked to the reduced potential to disseminate via the blood stream. miRNAs have been widely associated with cancer stem cell phenotype in colorectal cancer mainly via regulating Wnt/B-catenin and Notch signalling [22, 23]. For instance, miR-215, which is among those miRNAs most often reported on in CSCs, has been demonstrated to regulate CSC differentiation and chemoresistance [23–26]. Search of miR-215 target genes revealed that miR-215 may repress expression of cell cycle and stemness genes while downregulation of miR-215 itself augments clonogenicity of colon cancer cells [24]. Interestingly, we found miR-215 (hsa-miR-215-5p) to be repressed in those primary tumors that metastasized to the liver as compared to those that spread to the peritoneum. This may support the cancer stem cell like phenotype of colorectal cancer metastasized to the liver as hypothesized in our former study [5].

In general, research on how miRNAs may be involved in determining metastatic spread is on the increase [27]. However, previous studies compared colorectal cancer tissue to normal non-cancer controls, drawing conclusions on those miRNAs potentially involved in the biology of CRC more generally [28–30]. To the best of our knowledge, no study has been published on how miRNAs may influence the route of metastasis i.e. via the blood stream into distant organs or via local spread into the peritoneum. The current study identified a couple of miRNAs to be differentially expressed among CRC samples that set metastasis into either liver or peritoneum and stage-matched CRC cases that had not set any metastasis at all. Several miRNAs identified in our analysis had already proven to be differentially expressed in colorectal cancer or may even be associated with clinico-pathological parameters [25, 27, 28]. Hence, our data may encourage functional analysis linking these miRNAs to metastasis formation in in vitro and in vivo models.

Beyond hsa-miR-215-5p another two miRNAs were validated on the whole patient panel. We selected hsa-miR-483-5p, since it was strongest repressed miRNA when expression of PER vs. LM was compared (Fig. 2b). In contrast, hsa-miR-31-5p was chosen since it was the second most obviously upregulated (regarding PER to LM, Fig. 2b) miRNA. hsa-miR-31-5p underwent functional analysis due to three reasons. When the analysis was extended on the whole patient sample, differential expression only remained significant in case of hsa-miR-31-5p (Fig. 3c). In addition, its star strand, i.e. hsa-miR-31-3p, was also differentially expressed, though failed to fulfil the criteria of triple differential expression. Interestingly, up-regulation of both hsa-miR-31-5p and its passenger strand hsa-miR-31-3p was detected to be predictive in metastatic CRC patients treated with cetuximab - again suggesting this miRNA to play a dominant role in metastatic CRC [31]. Finally, miR-31 has been demonstrated to regulate cMet in ovarian cancer [15].

Of course, demonstrating inverse correlation of miR-31 and c-MET at the exact time point (!) of metastatic spread would be desirable. However, about half of our primary tumor samples were resected years before metastatic spread was diagnosed (i.e. metachronous metastatic spread) and thus our samples did not mirror the state or time point of metastatic spread. Since both metastasis formation and miRNA mediated gene regulation are highly dynamic processes, determination of c-MET at surgery of the primary tumor, i.e. years before metastasis formation may take place, seems not to be useful in terms of analyzing whether miR-31 may regulate c-MET. This may explain why there was no significant statistical association of miR-31 and c-MET immunopositivity in our tumor samples. An additional reason for this is that initiation of metastasis formation is a complex process and is supposed to be restricted to stem cells or small sub-populations within the tumor [32–34]. Hypothetically assuming that miR-31 regulates c-MET in cancer cells thus to participate in determining metastatic spread, such a regulation process may only take place in single clones that initiate metastasis formation. The method used to determine c-MET (= single marker IHC) in the current study was not suited to discriminate metastasis initiating sub-clones from the rest of the tumor cells. Hence, we quantified c-MET throughout the whole tumor sample rather than in specific sub-populations. This may methodically mask potential regulatory effects of miR-31 on c-MET in certain sub-populations of tumor cells.

### Hsa-miR-31-5p may act on epithelial-to-mesenchymal transition by target gene regulation

Epithelial-to-mesenchymal transition (EMT) is regarded to be a major step within metastasis formation [19]. miR-31 has been demonstrated to regulate a number of genes closely associated with EMT and metastasis formation. For instance, miR-31 was found to inhibit SATB2 thereby supporting CRC cell growth, invasion and metastasis formation [16]. On the other hand, miR-31 overexpression has been shown to inhibit autophagy in cancer associated fibroblasts of CRC thereby increasing apoptosis and reducing migration of co-cultured CRC cell lines [17]. Besides this observation, autophagy rate may also serve as a readout parameter for miR-31 activity – even in FFPE samples. Induction of miR-31 inhibited integrin alpha V and thus reduced metastatic potential of gastric cancer cells [18]. In line with this, several authors already highlighted miR-31 to directly target c-MET (also known as scatter factor) - a prominent mediator of EMT [15, 35, 36]. The current work was able to reproduce this finding in colon cancer cells. Hence, it may be hypothesized that repression of hsa-miR-31-5p in LE samples may lead to up-regulation of c-MET – at least at the time of initial metastatic spread. Whether repression of c-MET by hsa-miR-31-5p may also support EMT and whether this may aid tumors cells to spread via the blood stream needs to be determined. Finally, the current study demonstrated induction of hsa-miR-31-5p in those primary tumors that spread to the peritoneum - again supporting the hypothesis that hsa-miR-31may regulate metastatic potential of CRC.

## Conclusion

This study found that miRNA expression of colorectal cancer primary tumor tissue may predict the site of synchronous or metachronous metastatic spread. We further identified hsa-miR-31-5p to be overexpressed in those CRC cases that were diagnosed with peritoneal carcinomatosis either at initial diagnosis or during follow up. Interestingly, miR31 has been shown to repress expression of c-MET [15] - a kinase known to play a pivotal role in epithelial-to-mesenchymal-transition and hematogenous metastatic spread [37, 38].

## Abbreviations

CRC: Colorectal cancer
EMT: Epithelial-to-mesenchymal transition
LM: Liver metastasis
M0: No distant metastasis
miRNA: micro RNA
PER: Peritoneal carcinomatosis
PVDF: Polyvinylidene fluoride
TLDA: TaqMan Low Density Array
